# Resonators with tailored optical path by cascaded-mode conversions

**DOI:** 10.1038/s41467-023-35956-9

**Published:** 2023-01-30

**Authors:** Vincent Ginis, Ileana-Cristina Benea-Chelmus, Jinsheng Lu, Marco Piccardo, Federico Capasso

**Affiliations:** 1 Harvard John A. Paulson School of Engineering and Applied Sciences, Harvard University, Cambridge, MA 02138 USA; 2 Data Lab / Applied Physics, Vrije Universiteit Brussel, 1050 Brussel, Belgium; 3 Hybrid Photonics Laboratory, École Polytechnique Fédérale de Lausanne, Lausanne, CH-1015 Switzerland; 4 Center for Nano Science and Technology, Istituto Italiano di Tecnologia, Milan, 20133 Italy

**Keywords:** Applied optics, Optical physics, Microresonators

## Abstract

Optical resonators enable the generation, manipulation, and storage of electromagnetic waves. The physics underlying their operation is determined by the interference of electromagnetic waves, giving rise to the resonance spectrum. This mechanism causes the limitations and trade-offs of resonator design, such as the fixed relationship between free spectral range, modal linewidth, and the resonator’s refractive index and size. Here, we introduce a new class of optical resonators, generating resonances by designing the optical path through transverse mode coupling in a cascaded process created by mode-converting mirrors. The generalized round-trip phase condition leads to resonator characteristics that are markedly different from Fabry-Perot resonators and can be tailored over a wide range. We confirm the existence of these modes experimentally in an integrated waveguide cavity with mode converters coupling transverse modes into one supermode. We also demonstrate a transverse mode-independent transmission and show that its engineered spectral properties agree with theoretical predictions.

## Abstract (web summary)

Resonators are key components in optics. In this work, the authors introduce a class of optical resonators with distinctly different properties from conventional resonators, allowing fundamental design trade-offs to be circumvented.

## Introduction

Optical resonators are a cornerstone of modern physics and technology^1–3^. These optical devices have two essential functions: they provide spectral selectivity to incident light and enhance its intensity in a small volume of space^4–6^. A prime example of a device that exploits both spectral selectivity and field amplification within a resonator is the laser, in essence, an optical cavity in which an active medium and a pumping mechanism are present^7^. In addition, resonators with an embedded crystalline nonlinearity enable efficient frequency doubling, sum and difference frequency generation, optical parametric amplification, and optical isolation^5,8–11^. The spectral sensitivity of resonators is also widely used in chemical, biological, and thermal spectroscopy, and in optical communication networks in filters, switches, and optical delay lines^12–14^. Furthermore, the transfer of momentum between light and matter^15^ can be enhanced inside a cavity, a feature widely used in cavity optomechanics. Optical resonators also provide an ideal platform to study and control quantum mechanical interactions^16–19^ and have played a key role in the development of quantum systems with ultrastrong coupling^20^. Several analogs of nonlinear-optics phenomena have been demonstrated by coupling two-level atoms with resonator modes^21^. Finally, collective phenomena arise in an array of coupled optical resonators, including an effective magnetic field for photons^22^, non-reciprocal phase shifts, and topologically protected edge states^23^, useful for unidirectional and robust guiding of light ^24–26^. Recent examples are the demonstrations of the mirror-symmetric non-reciprocal circulators^27,28^ and the time-multiplexed photonic resonators with isolated dissipation rates and dissipation spectra with non-trivial topological invariants^29^.

Motivated by this multitude of applications, various innovations have been devised to design the properties of resonators. One approach uses photonic crystals to modify the propagation constants inside the medium^30^. By exploiting the unusual properties of epsilon-near-zero media, geometry-invariant resonant cavities have been demonstrated^31^. Nonlinear processes, such as second- and third-harmonic generation, have been optimized inside carefully engineered ring resonators based on silicon nitride (Si3N4) and silicon dioxide (SiO_2_)^32^ and a SiN-2D hybrid platform has been introduced to overcome the amplitude-phase trade-off in ring resonators^33^. Most recently, various novel cavity implementations have been realized where nanostructured mirrors are engineered to manipulate the cavity’s phase shift. This approach allows the cavity length to be reduced^34^. This same technique can be used to construct stable plano-planar cavities^35^ and cavities with engineered transmitted beam profiles^36,37^. Finally, there is a strong research interest in the design and application of resonant bound states in the continuum^38^, localized modes, coexisting with a continuous spectrum of radiating waves, because of their potential to realize high-*Q* dynamic resonances^39^, miniaturize nano resonators^40^, and create vector beams^41^.

In this work, we introduce cascaded-mode resonators. The functionality of these resonators is based on cascaded-mode coupling between different transverse modes^42^.

## Results

### Theory of cascaded-mode resonances

**Fig. 1:**
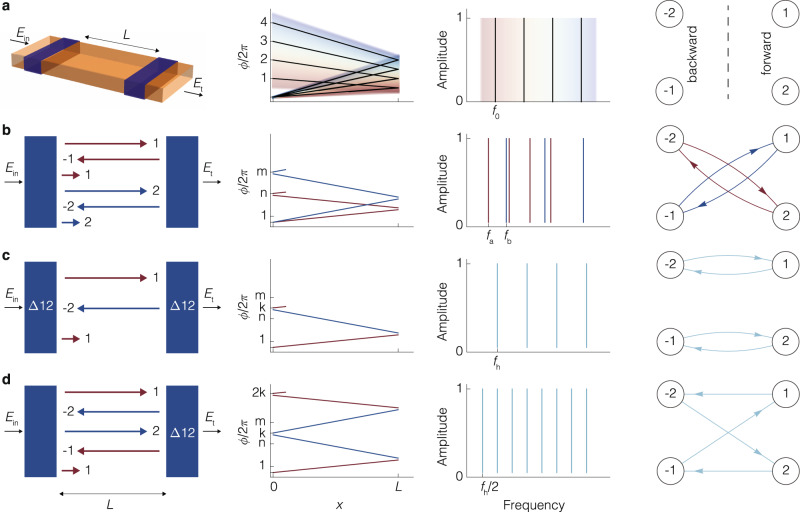
The operating principle underlying cascaded-mode resonances. **a** From left to right: First, a visualization of traditional resonator of length *L*. Second, the phase shift as a function of distance *x* for a resonator of length *L* for different longitudinal modes of index *m*: after a round trip 2*L* the accumulated phase is *m*2*π*. Third, the resonance spectrum, which corresponds to the frequencies for which the round-trip phase Δ*ϕ* equals a multiple of 2*π*. **b** In many resonators, different transverse modes contribute to different spectra because their effective refractive indices inside the resonator differ. The different effective indices determine the different slopes of the lines in the second column, which results in spectra with different fundamental modes (*f*_a_, *f*_b_) and mode spacings in the third column. **c** A cascaded-mode resonator (the blue regions are mode-converting mirrors) in which the two transverse modes, labeled 1 and 2, couple into one supermode, with fundamental frequency *f*_h_. The round-trip phase and the free spectral range are partly determined by the effective indices of mode 1 (red slope) and of mode 2 (blue slope). **d** A cascaded-mode resonator in which a supermode is created where both mode 1 and mode 2 circulate twice through the resonator before completing the round trip. There is one spectrum, with fundamental mode and free spectral range halved compared to **c**. The labels in the resonators in the first column refer to the mode conversions that take place in the blue regions: Δ12 implies that mode 1 is reflected into mode 2, and vice versa. Blue regions without label refer to traditional mirrors where each mode is reflected into itself. The fourth column illustrates the directed graph description of each resonator. In this representation, the vertices correspond to the modes and the lines between the nodes correspond to the different mode converters. The loops in these graphs identify the different resonances.

The functionality of electromagnetic resonators can be understood from the constructive interference of waves—creating resonant modes. A crucial parameter that determines these modes is the round-trip phase Δ*ϕ*, accumulated by the field after completing one round trip in the resonator^2^. Waves that pick up a round-trip phase equal to a multiple of 2*π* constructively interfere with themselves and become resonant modes of the resonator (Fig. 1a). In the case of a Fabry-Perot geometry, the resonance condition is then given by

1 $$2Ln\frac{2\pi \nu }{c}+2{\phi }_{r}=2\pi m,$$

where *ν* is the frequency of light, *m* is an integer number representing the index of the resonant modes of frequency *ν*_*m*_, *c* is the speed of light in vacuum, *L* is the length of the resonator, *n* is the refractive index of the material inside the resonator, and *ϕ*_*r*_ is the reflection phase at the mirrors. This simple equation explains two essential properties of resonators: the existence of the fundamental mode and the appearance of a spectrum with only a discrete number of modes. The resulting frequency spectrum from Eq. (1) is then given by *ν*_*m*_ = *c*(*m* − *ϕ*_*r*_/*π*)/(2*nL*).

Above, we ignore the properties of the mode in the transversal plane. Typically, a discrete number of orthogonal transverse modes exist for each frequency, e.g., TE_*i*_ and TM_*i*_ waves, where each transverse mode experiences a different effective index (*n*_eff,*i*_). As a result, the resonant modes of a resonator generally consist of a superposition of spectra, corresponding to the various families of transverse modes (Fig. 1b). The spectra are given by

2 $${\nu }_{i,m}=\frac{c(m-{\phi }_{r,i}/\pi )}{2{n}_{{{{{{{{\rm{eff}}}}}}}},i}L}.$$

We now introduce a new type of resonator based on cascaded-mode coupling. This coupling is implemented by mode-converting mirrors that not only reflect incident waves, but also simultaneously convert them to another transverse mode profile^42^. We illustrate this principle in Fig. 1c, d: upon reflection on the rightmost mode-converting mirror, an incident wave with a particular transverse mode profile is converted into another transverse mode. When this mode returns to the leftmost mirror, another mode conversion occurs upon reflection. This cascade of mode conversions can be repeated as many times as the number of transverse modes supported by the waveguide. Finally, a “supermode” emerges when the wave is converted back to the original configuration of the incident mode. For resonators with *N* different transverse modes, the round-trip phase is given by (Supplementary Materials):

3 $$\Delta \phi={k}_{0}L\xi \mathop{\sum }\limits_{i=1}^{N}{n}_{{{{{{{{\rm{eff}}}}}}}},i}+{\phi }_{r,{{{{{{{\rm{tot}}}}}}}}}.$$

Here *k*_0_ equals 2*π*/*λ*_0_ with *λ*_0_ the vacuum wavelength, *ϕ*_*r*,tot_ is the sum of all reflection phases, and *ξ* is the parameter that encodes whether the contributing transverse modes appear once (*ξ* = 1) or twice (*ξ* = 2) in the chain. The round-trip phase is thus no longer merely determined by the length of the resonator and the refractive index but also by the number of coupled transverse modes. The corresponding resonance condition is

4 $${\nu }_{m}=\frac{c\left[m-{\phi }_{r,{{{{{{{\rm{tot}}}}}}}}}/(2\pi )\right]}{L\xi \mathop{\sum }\nolimits_{i=1}^{N}{n}_{{{{{{{{\rm{eff}}}}}}}},i}}.$$

The free spectral range is thus set by the sum of the round-trip optical paths of the different cascaded modes $L\xi \mathop{\sum }\nolimits_{i=1}^{N}{n}_{{{{{{{{\rm{eff}}}}}}}},i}$ rather than by 2*n*_eff,*i*_*L* as in a conventional resonator. Next, whereas traditional resonators feature an incoherent superposition of different spectra, each corresponding to a different transverse mode, cascaded-mode resonators exhibit just one superspectrum (Fig. 1c, d).

This analysis is independent of how the mode conversions are realized. For instance, in the context of transverse modes in waveguides, a mode converter can be implemented using a specific refractive index variation (blue regions in Fig. 1a–d). The last column of Fig. 1 presents a useful abstraction to visualize and study cascaded-mode resonances using directed graphs. In this picture, cascade-mode resonances appear as cyclic graphs, which allows for studying the resonators using the properties of their associated adjacency matrix. (Supplementary Materials).

Above, we only consider the round-trip phase resonance condition to get insights into the spectrum of cascaded-mode resonators. To obtain a more accurate picture of this spectrum, we need to account for both the phase and the amplitude of the different waves. The transmission spectrum **E**_out_ of a cascaded-mode resonator, where *N* different forward-propagating modes are coupled with each other, is given by (Supplemental Material):

5 $${{{{{{{{\bf{E}}}}}}}}}_{{{{{{{{\rm{out}}}}}}}}}=\mathop{\sum }\limits_{i=1}^{N}\frac{{t}_{{{{{{{{{\rm{pt}}}}}}}}}_{i}}{{{{{{{{\rm{e}}}}}}}}}^{{{{{{{{\rm{i}}}}}}}}{\phi }_{i}}}{1-{r}_{{{{{{{{\rm{rt}}}}}}}}}{{{{{{{{\rm{e}}}}}}}}}^{{{{{{{{\rm{i}}}}}}}}\Delta \phi }}{E}_{{{{{{{{\rm{in}}}}}}}}}{{{{{{{{\bf{u}}}}}}}}}_{{{{{{{{{\rm{f}}}}}}}}}_{i}}.$$

Here *r*_rt_, ${t}_{{{{{{{{{\rm{pt}}}}}}}}}_{i}}$, *ϕ*_*i*_, and ${{{{{{{{\bf{u}}}}}}}}}_{{{{{{{{{\rm{f}}}}}}}}}_{i}}$ are respectively the round-trip reflection coefficient, the pass-through transmission amplitude, the transmission phase, and the unit vector of the forward propagating mode *i* (Supplemental Materials).

**Fig. 2:**
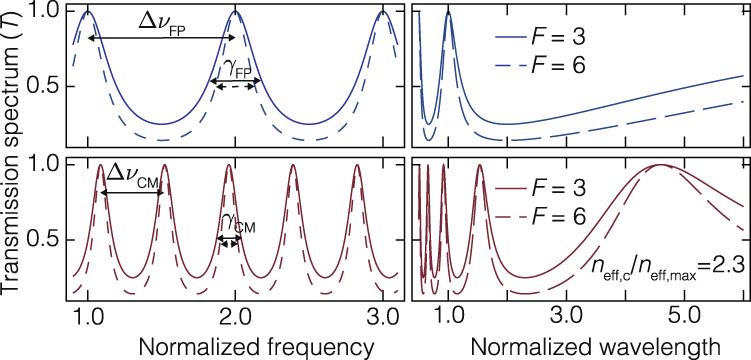
The spectral properties of cascaded-mode resonances. The spectra of a traditional resonator compared with the spectra of a cascaded-mode resonator versus frequency (left) or wavelength (right). The blue and red spectra respectively correspond to conventional resonators and cascaded-mode resonators with ${n}_{{{{{{{{\rm{eff,c}}}}}}}}}/{n}_{{{{{{{{\rm{eff,max}}}}}}}}}=2.3,{\phi }_{{{{{{{{\rm{r,tot}}}}}}}}}=1.5\pi$. The solid and dashed lines correspond to a resonator’s finesse equal to 3 and 6, respectively. Note the reduction of the free spectral range Δ*ν* and linewidth *γ* when several transverse modes are coupled.

An interesting feature of cascaded-mode resonances, in agreement with the geometrical model described above, is the modification of the free spectral range Δ*ν*, given by

6 $$\Delta \nu=\frac{c}{\xi \mathop{\sum }\nolimits_{i=1}^{N}{n}_{{{{{{{{\rm{g}}}}}}}},i}L},$$

where *n*_g,*i*_ is the group index of transverse mode *i* at frequency *ν*. Two other crucial spectral parameters can be engineered in a cascaded-mode resonator by controlling the round-trip phase: the linewidth *γ* and the quality factor *Q* (Fig. 2). Unlike the free spectral range, the linewidth and the quality factor depend on the round-trip losses (Supplementary Materials).

Not only the spectral properties but also the temporal and spatial properties of these modes can be engineered by using cascaded-mode coupling. The intracavity power build-up and the intracavity power build-up time both scale proportionally to the number of coupled modes. While the intensity of longitudinal modes in traditional resonators exhibits a simple standing-wave profile, the intensity profile in a cascaded-mode resonator will have a more irregular profile, potentially with many different local minima and maxima.

A unique spatial property of cascaded-mode resonators is that the propagation constant of a supermode depends on the propagation direction. This phenomenon is shown in its most straightforward implementation in Fig. 1c. When a field with transverse profile of mode 1 is incident on the left side of this resonator, a cascaded mode will exist with wave vector *k* = *k*_0_*n*_eff,1_ propagating from left to right, and a wave vector *k* = *k*_0_*n*_eff,2_ propagating from right to left. Due to the distinct propagation constants in opposite directions, directional nonlinear optical effects can occur in the resonator since the phase-matching conditions may only be satisfied in one direction^43^. The directionality could also give additional control over chiral, optomechanical, or quantum mechanical interactions inside the resonator.

A final property of cascaded-mode resonances that deserves special attention is the existence of mode-independent spectra. Indeed, different transverse modes at the input may excite the same resonance, i.e., a mode-independent resonance. As an example, in the resonators of Fig. 1c–d the transmission spectrum (third column) is the same for the two incident transverse modes (1 and 2). We show in Supplementary Materials that the different modes that excite the same resonance in a cascaded-mode resonator can be extracted from the adjacency matrix of the graph that encodes the different mode conversions in the resonator. The mode-independent behavior of cascaded-mode resonators is a unique transmission characteristic, a feature verified experimentally in Fig. 4. This is in contrast to traditional resonators, where different transverse modes exhibit different transmission spectra. Based on this property, it becomes possible to manipulate modes with different spatial profiles in an identical way using only one resonator.

### Experiments

We experimentally realize the proposed cascaded-mode resonators using the silicon-on-insulator (SOI) platform at telecom wavelengths (1550 nm). In our on-chip implementation, the cascaded modes have distinct transverse profiles TE_*i*_, an in-plane polarization, and propagate along waveguides rather than in free space. The SOI platform offers design flexibility in engineering the properties of the mode converters (reflection phase and magnitude), as well as the propagation properties of all modes participating in the cascade, such as their effective indices *n*_eff,*i*_.

**Fig. 3:**
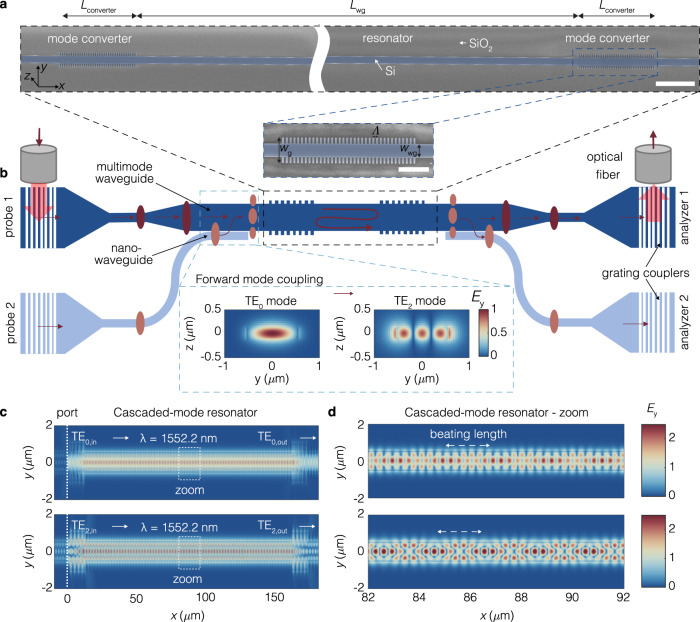
The experimental realization of cascaded-mode resonators in integrated photonics. **a** SEM pictures of the cascaded-mode resonator show two mode converters connected via a multimode waveguide of width *w*_wg_ and length *L*_wg_. Multimode waveguides located before and after the resonator guide telecom light into and outside the resonator. The mode converters are realized by corrugating the silicon waveguide laterally into the shape of a rectangular grating of periodicity Λ and width *w*_g_. Scalebar = 5 *μ*m and 2 *μ*m (inset). The periodicity Λ is chosen such that the phase-matching condition is satisfied for contra-directional coupling. The entire photonic circuit ridge is buried into a silica layer. **b** Schematic of the device shows three different sections: 1. two input waveguides (left) that allow to probe the resonator with either TE_0_ (upper) or TE_2_ (lower), 2. the resonator region consisting of the multimode waveguide enclosed by the two mode converters, and 3. two analyzer waveguides which transmit the output of the resonator into two spatially separated locations, depending on its transverse profile TE_0_ (upper) or TE_2_ (lower). Probe 1 excites the TE_0_ mode in the top waveguide. Probe 2 excites the TE_0_ in the lower waveguide. This mode is converted into the TE_2_ mode in the top multimode waveguide prior to the resonator via the forward-mode coupler, which operates on the principle that the effective index of the TE_0_ mode in the nano-waveguide corresponds to the effective index of the TE_2_ mode in the multimode waveguide. Similarly analyzer 1 and analyzer 2 measure TE_0_ and TE_2_ modes, respectively. Spatially, the coupling occurs at the location where the nanowaveguide is in the immediate vicinity of the multimode waveguide. **c** Full-wave simulations of the telecom fields inside the cascaded-mode resonator demonstrate that self-consistent solutions of the round-trip condition occur at the same input wavelength for two distinct transverse modes TE_0_ (upper) and TE_2_ (lower). **d** Zoom into marked white region inside the resonator reveals the hybrid nature of the cascaded modes that arise as a superposition of counter-propagating TE_0_ or TE_2_ modes with a characteristic beating length that does not depend on the input probe field.

The device geometry is shown in Fig. 3a, b together with scanning electron microscope (SEM) pictures of the fabricated structures. Further details are provided in the Supplementary Materials. In general, each device consists of three main optical components: input/output waveguides that couple and guide light of chosen transverse modes to and away from the mode-converting resonators; a multi-mode waveguide section of length *L*_wg_ in which the cascaded modes are confined; specialized corrugated Bragg reflectors located on either side of the multi-mode waveguide that reflect one transverse mode into another. While, as described theoretically above, the number of conversions in a cascaded mode is only limited by the number of available transverse modes, we restrict our experimental demonstration to cascaded-mode resonators of the type shown in Fig. 1c that couple the two distinct transverse modes TE_0_ and TE_2_. Their transverse mode profiles are shown in the inset of Fig. 3b. Consequently, the width of the waveguide in the cavity region (*w*_wg_ = 1.07 *μ*m) was chosen such that it cuts off all transverse modes of a higher order than TE_2_. (See Supplementary Materials for details on the design of the individual photonic structures and their transmission/filter performance.) In addition, the grating period Λ of the mode converters is chosen as to satisfy the phase-matching condition and provide the necessary momentum for the mode conversion to occur on the reflected wave: 2*π*/Λ = Δ*β*_12_ = *β*_1_ + *β*_2_, with ${\beta }_{1}={\beta }_{{{{{{{{{\rm{TE}}}}}}}}}_{0}}={n}_{{{{{{{{{\rm{eff,TE}}}}}}}}}_{0}}{\omega }_{0}/c$ and ${\beta }_{2}={\beta }_{{{{{{{{{\rm{TE}}}}}}}}}_{2}}={n}_{{{{{{{{{\rm{eff,TE}}}}}}}}}_{2}}{\omega }_{0}/c$ the propagation constants of the two coupled modes. This type of coupling is typically referred to as contra-directional coupling. In the Methods, we outline in more detail the strategy for designing the cascaded-mode resonators in the SOI platform.

**Fig. 4:**
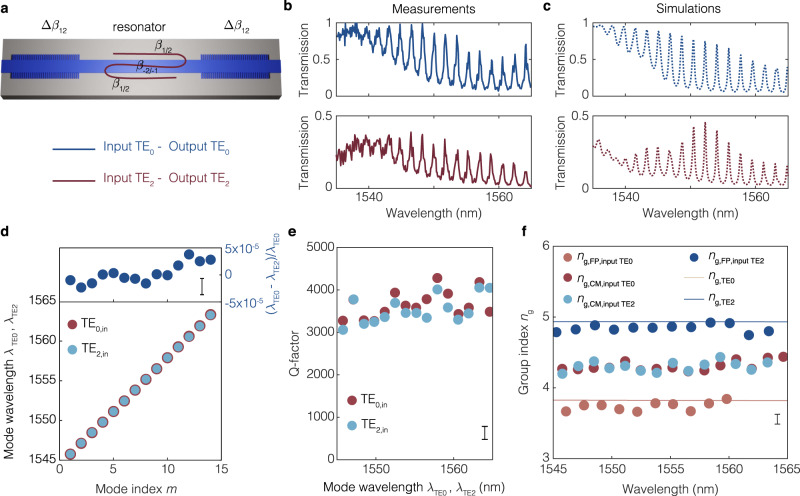
Transmission spectroscopy of cascaded-mode resonators. **a** A cascaded-mode resonator, where a reflection at both the left and the right Bragg mirror results into a conversion of the transverse mode from TE_0_ to TE_2_ and vice-versa. **b** The measured transmission spectra of the cascaded-mode resonator exhibit resonances regardless of whether TE_0_ or TE_2_ is incident onto the resonator. **c** Simulated transmission spectra reproduce well the measurements. More measurement and simulation results are provided in the Supplementary Material. **d**–**f** Characteristic parameters of the cascaded-mode resonator extracted from the experimental transmission spectra. **d** The mode-independent character of cascaded-mode resonators is demonstrated by the fact that the resonant wavelengths of the cavity modes coincide in frequency (and wavelength), regardless of whether the resonator is probed by a TE_0_ or TE_2_ mode. The relative experimental mismatch between their resonant wavelengths is below 4 × 10^−5^, for all 13 considered modes, which is comparable to the experimental noise (upper panel). **e** Also the quality factors of the cascaded-mode resonator are mode-independent, as they coincide within the experimental error, regardless of whether the cascaded modes are excited by TE_0_ or TE_2_ inputs. The experimental error is calculated from the error of the fit of the resonance peaks. **f** In cascaded-mode resonators, the propagation constants change magnitude at each reflection off a mode-converting Bragg mirror, and the group indices of cascaded-mode (CM) resonances are equal to the arithmetic mean of conventional Fabry-Perot (FP) resonances in the same multimode waveguide. This property is confirmed experimentally, with TE_0_ modes having a group index of 3.75, TE_2_ modes having a group index of 4.85 and cascaded modes having a group index of 4.3. Full lines represent calculated group indices from full-wave simulations. Vertical bars represent error bars.

We now demonstrate in experiments and simulations the most evident signatures of cascaded-mode resonanators: the mode-independent spectrum with modified spectral parameters. The symmetric cascaded-mode resonator of Fig. 1c provides resonant confinement to input modes that correspond to either TE_0_ or TE_2_ transverse modes and has the same transmission spectrum for either input. We confirm this computationally in Fig. 3c, where we report the simulated field profile of the same cascaded-mode resonator for the two possible inputs and find a locally enhanced field inside the resonator in both cases. Moreover, the hybrid nature of the near-infrared cascaded mode inside the resonator becomes apparent in the zoom-in of the spatial profile shown in Fig. 3d. The field profile can be decomposed into a superposition of counter-propagating TE_0_ and TE_2_ waveguide modes that exhibit, as expected, the same beating length for both inputs (marked by the white arrow). We demonstrate this property experimentally by transmission spectroscopy and contrast it with two test Fabry-Perot resonators that employ standard mirrors and provide cavity confinement to only one of TE_0_ or TE_2_ modes. The experimental results are shown for the three cases in Fig. 4a–c and Supplemental Fig. S9: We find that cavity modes appear, as expected, for both TE_0_ and TE_2_ modes in the case of the cascaded-mode resonator only. Moreover, the experimental results are well-reproduced by our simulations. Cavity modes appear only for one of the two modes for the conventional Fabry-Perot resonators, while light is simply transmitted for the other modes. In the Methods, we describe the spectroscopic technique used in these measurements.

Next, we analyze the resonator properties of the cavity modes associated with the cascaded-mode resonators compared to the conventional Fabry-Perot modes in Fig. 4d–f. Firstly, we show in Fig. 4d that the intra-cavity modes of the cascaded-mode resonators excited by the two inputs (TE_0_ or TE_2_) coincide in frequency. We experimentally find a negligible relative deviation between the two sets of resonant wavelengths of $({\lambda }_{{{{{{{{{\rm{TE}}}}}}}}}_{0}}-{\lambda }_{{{{{{{{\rm{TE}}}}}}}},2})/{\lambda }_{{{{{{{{{\rm{TE}}}}}}}}}_{0}} \, \approx \, 4 \, {{{{{{{\rm{x}}}}}}}} \, 1{0}^{-5}$. Furthermore, the quality factors of the two sets are approximately equal, as shown in Fig. 4e. Finally, the group index of the cascaded modes is approximately equal to *n*_g_ = 4.3, regardless of whether they are excited by TE_0_ or TE_2_. In contrast, the group index of the Fabry-Perot modes are equal to ${n}_{{{{{{{{{\rm{g,TE}}}}}}}}}_{0}}=3.75$ and ${n}_{{{{{{{{{\rm{g,TE}}}}}}}}}_{2}}=4.85$ (Fig. 4f). This result confirms once more the cascaded-mode character of the measured spectra, particularly because the group index is approximately the arithmetic mean of the group indices of the participating transverse modes, in agreement with Eq. (7).

## Discussion

This work shows how electromagnetic resonators can be generalized to cascaded-mode resonators, where the spectrum of supermodes reflects the generalized round-trip phase condition of a cascade of different transverse modes propagating in different directions. The theory is generally valid for any cascade of orthogonal modes inside cavities of arbitrary shape and is thus not only applicable to a cascade of transverse mode profiles of an integrated waveguide^44^. Indeed, for the round trip to occur after *N* conversions, the *N* + 1-th mode in the chain needs to be indistinguishable from the first, i.e., with identical frequency, temporal shape, *k*-vector, polarization, and phase profile^45–47^. As such, the mechanism can also be applied in the context of ring resonators. In that case, interestingly, only one mode converter needs to be materialized. There are also opportunities to apply the concept in free-space setups. Here, the OAM dimension provides a natural basis of different modes that can be coupled with each other.

Key design parameters of traditional optical resonators are the length of the resonator and the refractive index of the material with the traditional resonance condition determined by the product of both parameters. For a given transmission or reflection spectrum, there is thus a trade-off between length and refractive index: one cannot reduce the length of the resonator without increasing the effective refractive index. Previous literature addresses this trade-off by either cleverly designing the effective index and group index of the medium (e.g., using photonic crystals) or by affecting the effective length of the cavity (e.g., using metasurface reflectors). We offer a different solution, which involves modifying the formula of the resonance condition itself. We circumvent the trade-off not by modifying the design parameters but by decoupling them at the level of the resonance condition itself. The spectral, temporal, and spatial properties are no longer solely determined by the length and refractive index of the medium, but also by the number of coupled modes. In addition, these resonators exhibit completely new properties not found in their traditional counterparts, e.g., mode-independent resonances and directionally dependent propagation properties. Moreover, we believe it is also interesting to investigate how the concept of this paper can be transposed to other electromagnetic resonances, e.g., singular plasmons^48^. Although the resonant spectrum of singular plasmon surfaces, which can be designed using transformation optics^49^ and implemented using periodically doped graphene sheets^50^, is quite different, here as well, different modes couple together, eventually collapsing the spectrum into a continuum. We anticipate that the concept of cascaded-mode resonators will be further exploited in a broad class of technological devices and scientific experiments since the underlying principles of cascaded-mode resonances can be extended even beyond optics.

## Methods

### Design of the cascaded-mode resonators

**Fig. 5:**
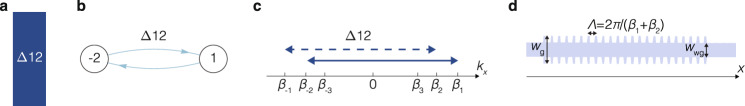
The different visualizations and design considerations of cascaded-mode resonators. **a** The visualization of a mode-converting mirror, reflecting mode 1 in mode -2, and vice-versa, used in the schematic in Fig. 1. **b** The equivalent graph representation of this mode-converting mirror, also used in Fig. 1. **c** In spatial frequency domain, the mode-converting mirror bridges the distance between the longitudinal *k*-components of the involved modes. A mode-converting mirror that converts mode *i* into −*j* (blue arrow) will also convert mode *j* to −*i*. (blue dashed arrow). **d** A potential, physical implementation of the mode-converting mirror in real space is a rectangular grating whose periodicity Λ is determined by the distance between the modes in *k*-space.

The design of a mode-converting mirror can be understood by visualizing the longitudinal *k*-components (*β*_*i*_) of the different guided modes in the waveguide (Fig. 5). A mode-converting mirror is a component that reflects one mode and simultaneously converts it into another mode. Therefore, they need to bridge a momentum equal to the difference between the propagation constants of both modes. A grating with periodicity Λ exhibits a principal spatial frequency component of 2*π*/Λ. In first order, the periodicity of the mode-converting mirrors is thus determined by ensuring that 2*π*/Λ = *β*_*i*_ + *β*_*j*_ for a grating that reflects mode *i* into mode − *j*. In the previous formula, we also used the identity *β*_−*j*_ = − *β*_*j*_. The phase-matching condition above is symmetric upon a permutation of *β*_1_ and *β*_2_, and the mode-converting grating is reciprocal under reflection of incident light with a transverse mode profile TE_0_ into TE_2_, and vice-versa. The selectivity of the mode converters also needs to be ensured: they need to reflect only the desired mode and not into any other modes. We note that the grating periodicity that satisfies the contra-directional coupling condition (Λ = 2*π*/(*β*_*i*_ + *β*_*j*_)) is much shorter than the one that meets the co-directional coupling condition (Λ = 2*π*/(*β*_*i*_ − *β*_*j*_)), in which case the converted mode would propagate in the same direction as the incident mode. Moreover, a reflection of the incident field into the same transverse mode (mode *i* into − *i* or *j* into − *j*) occurs if the grating periodicity Λ satisfies 2*π*/Λ = 2*β*_*i*_ or 2*π*/Λ = 2*β*_*j*_. The selectivity of the grating is determined by two elements: (1) the width of the principal spatial frequency of the grating, which is typically determined by the grating length, and (2) the difference between the propagation constants between of the different waveguide modes. Indeed, to selectively satisfy only one of these phase-matching conditions and selectively convert only the desired modes, these periodicities need to be sufficiently different from each other. As a result, if possible, a second design target is to engineer the effective indices of the two coupled modes to be as different as possible. This property is directly linked to the transverse dimensions of the waveguide. The design principles described above are based on a simple Fourier transform picture useful in the initial design of a mode-converting mirror. However, in any practical design, it remains important to supplement these principles with optimization based on numerical simulations, e.g., because the argument above is ignoring wave leakage as it propagates through the grating. The full-wave numerical simulations are shown in Supplemental Materials. We adopt a rectangular grating geometry that is symmetric with respect to the center of the ridge of the waveguide, has a periodicity Λ and a duty cycle of 40 % as shown in Fig. 3a. The waveguide width in the corrugated area *w*_g_ is larger than the waveguide width in between the reflectors *w*_wg_. At the same time, we need to satisfy low propagation loss and good confinement of the TE_2_ mode to the waveguide core. As shown in Fig. S4a, and in the mode profile of Fig. 3, a waveguide width of *w*_wg_ = 1.07 μm provides an index difference of ${n}_{{{{{{{{{\rm{eff,TE}}}}}}}}}_{0}}-{n}_{{{{{{{{{\rm{eff,TE}}}}}}}}}_{2}}=2.751-1.944=0.807$. In Fig. S8a–c we show the simulated mode conversion efficiency of the Bragg gratings used in the cascaded-mode resonator, in comparison with two test resonators which do not employ mode conversion but instead use standard Bragg mirrors that either reflect TE_0_ into TE_0_ or TE_2_ into TE_2_.

### Spectroscopic technique

The spectral response of each resonator is investigated under incident light that is either prepared to be in the TE_0_ or TE_2_ transverse mode. At first, the light is directed from an optical fiber placed above the chip into low-loss single-mode waveguides via grating couplers and adiabatic tapers. The single-mode waveguides filter any undesired higher-order transverse modes that may be excited by the grating couplers. Finally, an adiabatic taper ensures a low-loss transmission of the TE_0_ mode in the top arm (Fig. 3b, dark blue) from the single-mode waveguide to the multimode waveguide that precedes the cascaded-mode resonator. Importantly, we pattern around the resonator two distinct input/output ports (Fig. 3b, dark and light blue), which allow for the preparation of the input states entering the resonators and analyzed states exiting the resonator to be either in the TE_0_ or TE_2_ transverse mode. To this end, if the light is incident in the bottom arm (Fig. 3b, light blue), we generate the TE_2_ mode from TE_0_ before the resonator using a co-directional evanescent coupler based on a nano-waveguide located next to the multimode waveguide^51^. This coupling is visualized in the inset of Fig. 3b. The TE_2_ mode is coherently excited by the TE_0_ mode. From Fig. S4a, we find that this condition is satisfied if the nano-waveguide width equals 336 nm. The full-wave simulated field in Fig. S5 demonstrates an efficient forward coupling with 70% transmission at an interaction length of *L*_int_ = 60 μm, as used in the experiment.

### Numerical simulations

Lumerical FDTD Solutions (v8.21) is used to simulate and design the mode-converting gratings and the resonant cavity composed of them. In the simulations, the thickness of the device layer of the silicon is 220 nm. The substrate is silicon oxide. A layer of silicon oxide on top with a thickness of 700 nm is applied to protect the silicon devices. The refractive index of the silicon and silicon oxide are 3.46 and 1.46, respectively. The waveguide has a width of 1100 nm, allowing for the existence of guided modes of TE_0_ and TE_2_ around a wavelength of 1550 nm. The mesh size is 25 nm.

The period, depth, and duty cycle of the gratings used for converting a forward TE_0_ mode to a backward propagating TE_2_ mode (and vice versa) is 316.5 nm, 500 nm, and 40%, respectively. In this case, the mode conversion efficiency reaches a maximum near 1550 nm wavelength. The sweeping range of wavelength and the number of unit cells in the grating is 1350–1750 nm and 1–50, respectively. We use the Mode Source Module in the Lumerical software to solve the eigenmodes in the waveguide and select the TE_0_ or TE_2_ mode as the input light source into the mode converter gratings. The transmitted and reflected electromagnetic (EM) fields are recorded after the 3D full-wave simulations. The mode expansions of the recorded EM fields are performed to obtain the transmitted and reflected power of the TE_0_ and TE_2_ modes.

The resonant cavity is formed by two sets of above mentioned mode converter gratings with a cavity length of 150 μm. The number of unit cells of the mode-converting mirror is chosen to be N_per_ = 36 considering both a high mode-conversion efficiency and good bandwidth. The simulation time is set to be 72 ps which is long enough to get an accurate result.

### The directed graph representation of cascaded-mode resonators

**Fig. 6:**
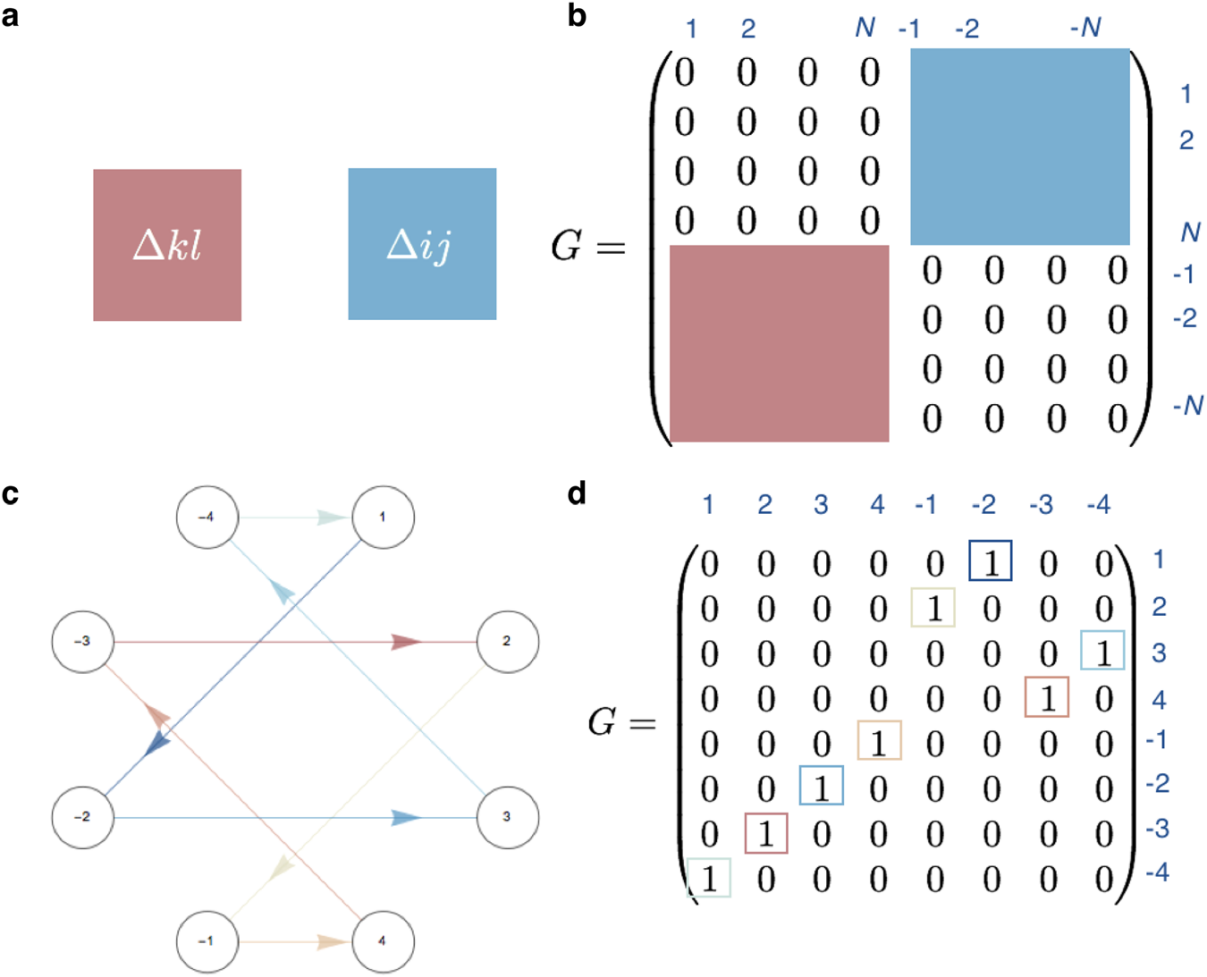
The directed graph representation of a general cascaded-mode resonator. **a** A schematic of the mode conversions in a cascaded-mode resonator. **b** The adjacency matrix of this cascaded mode resonator contains two symmetric submatrices that correspond to the left and right mode converters. **c** An example of the graph in a four-mode system where the left and right converter implement Δ23 + Δ14 and Δ12 + Δ34, respectively. **d** The adjacency matrix of the system shown in **c**.

We now discuss the relationship between the resonators, the mode converters, and the directed graphs in more detail. As shown in Fig. 6a, cascaded-mode resonators consist of two sets of converters that convert forward-propagating modes into backward-propagating modes and vice-versa. One can construct the directed graph of a cascaded-mode resonator by associating each mode with a node and each mode converter with an edge between the nodes. In doing so, it is essential to disambiguate the forward and backward propagating modes. Indeed, since there are no conversions between forward propagating modes or backward propagating modes, this procedure results in constructing a bipartite graph. The graph is directed since the mode conversions occur at one end of the resonator, and both ends of the resonator are not necessarily identical.

The general form of the adjacency matrix is shown in Fig. 6b. The action of the converters at both ends of the resonator is visible as separate submatrices in this matrix. Here, the color of the submatrices corresponds to the color of the converters. It is now worth mentioning a subtlety about the internal symmetry of the adjacency matrix. Since both mode converters can be significantly different for one another, the adjacency matrix is not symmetric. However, the mode converters themselves are generally symmetric. For example, if a converter converts mode i to mode −j, it generally also converts mode j to mode −i. Therefore, the submatrices that implement the two converters are, in turn, symmetric matrices.

In Fig. 6c–d, we show, by way of illustration, the graph of a specific cascaded-mode resonator where the right-hand converter consists of Δ12 + Δ34 and the left-hand converter consists of Δ23 + Δ14. The graph is shown in Fig. 6c, where each mode conversion has a different color. In Fig. 6d, we show the corresponding adjacency matrix where each element is circled by the color of the corresponding edge in the graph.

### Graph representation of mode-independent resonances

**Fig. 7:**
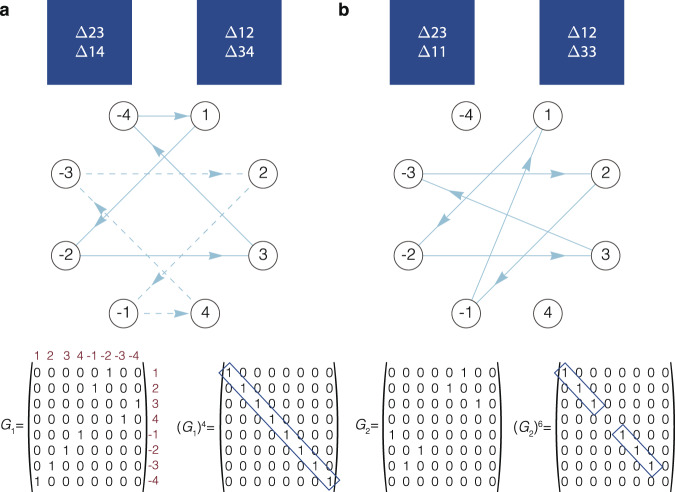
The directed graph representation to analyze mode-independent resonances in cascaded-mode resonators. **a** Top: a cascaded-mode resonator that couples four modes, where each mode appears only once in the resonance (*N* = 4, *ξ* = 1). Middle: the corresponding graph of the resonator contains two cycles. The two cycles (solid lines, dashed lines) exist because of the symmetry of the mode converters: if the edge *i* → −*j* exists, then the edge *j* → −*i* also exists. Bottom: The adjacency matrix of the graph (*G*_1_) and the adjacency matrix raised to the power 4, ${({G}_{1})}^{4}$. The adjacency matrix raised to the power *N* is a diagonal matrix, illustrating that a node is mapped back onto itself after a path of length *N*, or a cycle of length *N* exists in the graph. Each row with a non-zero element on the diagonal corresponds to a transversal mode that excites a mode-independent resonance. If several cascade-mode resonances of length *N* exist in one resonator, these modes can be separated in the adjacency matrix by weighting the edges by the propagation constants of the connecting nodes. **b** A cascaded-mode resonator with three coupled modes, each appearing twice in the resonance (*N* = 3, *ξ* = 2). The adjacency matrix (*G*_2_) is diagonalized after raising the matrix to the power 6 (=*ξN*).

There are generally two ways in which mode-independent resonances can appear in a cascaded-mode resonator. These two alternatives can be most easily understood by looking at the graph representation of the resonator, as shown in Fig. 7. As described earlier, we can recognize the resonances as the loops in the directed graph. To identify the mode-independent resonances, we can write out the different loops as a sequence of the vertices.

Mode-independent resonances can then occur within one loop or between two different loops. First, within one loop, the different nodes of the sequence all contribute to the same supermode. The resonator will thus experience mode-independent resonances for each of these modes as inputs. In addition, also two different loops can give rise to the same resonance. This is the case if the sequence of one loop can be turned into the sequence of the other by inversion of the nodes and reversing the direction of the sequence. The two alternatives are shown in Fig. 7. In Fig. 7b, e.g., there are mode-independent resonances for input 1, input 2, and input 3: indeed, {1, −2, 3, −3, 2, −1} is equivalent to {2, −1, 1, −2, 3, −3} and {3, −3, 2, −1, 1, −2}. In the case of Fig. 7a there are two loops in the graph: {1, −2, 3, −4} (solid lines) and {4, −3, 2, −1} (dashed lines). These loops are identical after inverting the nodes and reversing the sequence of one the loops. Incidentally, the two alternatives correspond to the parameter *ξ* being 1, in Fig. 7a, or 2, in Fig. 7b.

The different modes that activate the same supermode can also be retrieved from the graph’s adjacency matrix. For example, when the adjacency matrix raised to a power *k* has non-zero diagonal elements for some rows, then all the rows with the same diagonal element correspond to transversal modes that excite the same mode-independent resonance, as shown in Fig. 7.

### The transmission spectrum of cascaded-mode resonators with residual back-reflection

It is also possible to analyze the properties of cascaded-mode resonators in the case where the reflectors also reflect part of the modes back into themselves. The transfer matrix formalism allows to describe this situation most elegantly. We illustrate the formalism below for a two-mode resonator, defining the transmission and reflection matrices of the first and second reflector, respectively with subscripts *m*, 1 and *m*, 2, as follows:

7 $${T}_{m,1}={T}_{m,2}=\left(\begin{array}{cc}t&0\\ 0&t\end{array}\right),$$

8 $${R}_{m,1}={R}_{m,2}=\left(\begin{array}{cc}r&\kappa \\ -{\kappa }^{*}&{r}^{*}\end{array}\right).$$

Here, we assumed that both mirrors are identical. Conservation of energy imposes the following condition on *r*, *t*, and *κ*: ∣*r*∣^2^ + ∣*t*∣^2^ + ∣*κ*∣^2 ^≤ 1.The case of perfect mode-converting mirrors is retrieved for *r* = 0. In addition, we also define the propagation matrix, *T*_prop_, that tracks how the different modes evolve upon propagation through the cavity:

9 $${T}_{{{{{{{{\rm{prop}}}}}}}}}=\left(\begin{array}{cc}{{{{{{{{\rm{e}}}}}}}}}^{{{{{{{{\rm{i}}}}}}}}{\beta }_{1}L}&0\\ 0&{{{{{{{{\rm{e}}}}}}}}}^{{{{{{{{\rm{i}}}}}}}}{\beta }_{2}L}\end{array}\right).$$

**Fig. 8:**
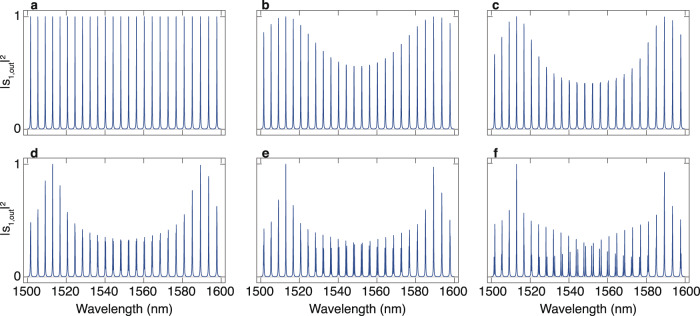
The transmission spectrum of cascaded-mode resonators with residual back-reflection. Visualization of (${\left|{s}_{1,{{{{{{{\rm{out}}}}}}}}}\right|}^{2}$) with **s**_in_ = $$\left(\begin{array}{c}1\\ 0\end{array}\right)$$. The reflection in panels **a**–**f**, equals *r* = 10^(−6)^, *r* = 0.001, *r* = 0.003, *r* = 0.01, *r* = 0.03, *r* = 0.1, respectively. Small values of *r* translate into an amplitude modulation of the spectrum. For large values of *r*, an observable hybridization of the modes appears. In each panel, the transmission of the mirrors, as defined in Eq. (7), equals $t=\sqrt{0.1}$, the length of the resonator equals *L* = 114.85 μm, *n*_eff,1_ = 2.74, and *n*_eff,2_ = 2.47. The mode-coupling constant is calculated using $\kappa=\sqrt{1-{t}^{2}-{r}^{2}}$.

Using these matrices, it is possible to calculate the transmitted field and the spectrum of the resonator for any combination of *r* and *κ*. The rows of the input vector **s**_in_ contain the amplitude of the modes incident on the resonator. The rows of the output vector **s**_out_ contain the amplitudes of the modes transmitted through the resonator. We can then write that:

10 $${{{{{{{{\bf{s}}}}}}}}}_{{{{{{{{\rm{out}}}}}}}}}=\mathop{\sum }\limits_{k=0}^{\infty }{T}_{m,2}{({T}_{{{{{{{{\rm{res}}}}}}}}})}^{k}{T}_{{{{{{{{\rm{prop}}}}}}}}}{T}_{m,1}{{{{{{{{\bf{s}}}}}}}}}_{{{{{{{{\rm{in}}}}}}}}},$$

where ${T}_{{{{{{{{\rm{res}}}}}}}}}={T}_{{{{{{{{\rm{prop}}}}}}}}}{R}_{m,1}{T}_{{{{{{{{\rm{prop}}}}}}}}}{R}_{m,2}$ is the resonator matrix. Each term in the infinite sum is associated with fields transmitted after an extra roundtrip through the resonator. Utilizing the fact that all eigenvalues of ${T}_{{{{{{{{\rm{res}}}}}}}}}$ are smaller than 1, we know that

11 $$\mathop{\lim }\limits_{k\to \infty }{({T}_{{{{{{{{\rm{res}}}}}}}}})}^{k}=0$$

and we can rewrite the infinite sum as:

12 $${{{{{{{{\bf{s}}}}}}}}}_{{{{{{{{\rm{out}}}}}}}}}={T}_{m,2}{(I-{T}_{{{{{{{{\rm{res}}}}}}}}})}^{-1}{T}_{{{{{{{{\rm{prop}}}}}}}}}{T}_{m,1}{{{{{{{{\bf{s}}}}}}}}}_{{{{{{{{\rm{in}}}}}}}}},$$

where *I* is the identity matrix. This equation allows to calculate the spectrum of a cascaded-mode resonator for different values of *κ*, *r*, and *t*. In Fig. 8, we evaluate Eq. (12) to illustrate the spectrum of ${\left|{s}_{1,{{{{{{{\rm{out}}}}}}}}}\right|}^{2}$ assuming mode 1 is incident at the input. For large values of *r*, mode hybridization occurs. Even in these cases, where *r* > 0, the spectrum remains independent of the incident mode.

## Supplementary information

Supplementary Information

## Data Availability

The data generated in this study have been deposited in the Zenodo database under accession code 7441921. The data are available under CC BY 4.0.
