# Supplementary material for: Resonators with tailored optical path by cascaded-mode conversions
Source: Nat Commun. 2023 Jan 30;14:495. doi: 10.1038/s41467-023-35956-9 (PMC9886910; doi:10.1038/s41467-023-35956-9)
Supplement: Supplementary file 1 — Supplementary Information [file 41467_2023_35956_MOESM1_ESM.pdf]

Supplementary Material for:
Resonators with tailored optical path by cascaded-mode conversions

Vincent Ginis,[1,2*] Ileana-Cristina Benea-Chelmus,[1,3*]
Jinsheng Lu,[1] Marco Piccardo,[1,4] and Federico Capasso[1]
[1]Harvard John A. Paulson School of Engineering and Applied Sciences, Harvard University
Cambridge, Massachusetts 02138, USA
[2]Data Lab / Applied Physics, Vrije Universiteit Brussel
1050 Brussel, Belgium
[3] Hybrid Photonics Laboratory, École Polytechnique Fédérale de Lausanne
Lausanne CH-1015, Switzerland
[4]Center for Nano Science and Technology, Istituto Italiano di Tecnologia
Milan 20133, Italy
[*]Authors contributed equally to this work
E-mail: ginis@seas.harvard.edu, capasso@seas.harvard.edu.

CONTENTS

I. Theory 2
A. Maximal round-trip phase in general cascaded-mode resonators 2
B. The transmission spectrum of cascaded-mode resonators 2
C. Examples of graphs and adjacency matrices related to cascaded-mode resonators 4
D. Summary of scaling of spectral properties in cascaded-mode resonators 6

II. Materials and Methods 6
A. Fabrication 6
B. Characterization setup 6
C. Extraction of experimental resonator parameters 7

III. Simulations and Experiments 8
A. Design of co-directional waveguide coupler 8
B. Design of contra-directional mode-converting and non-mode converting Bragg gratings 9
C. Cascaded-mode resonators and Fabry-Perot resonators 9
D. The extraction of the experimental resonator parameters 10

References 13

I. THEORY

A. Maximal round-trip phase in general cascaded-mode resonators

We calculate the maximal round-trip phase that can be obtained in a cavity of length L with N different transverse modes, each experiencing an effective index $n_{\mathrm{eff},i}$. A central observation in this derivation is the maximum number of times a transverse mode can be excited in one round trip. Each transverse mode can occur only two times in the round trip: once in each direction. Indeed, each mode can only be coupled with another mode in the left and right ends of the resonator. In general, mode i will be coupled to mode j on the left end through the conversion Δij, and i will be coupled to k on the right end through the conversion Δik. Adding an extra conversion on either side will split the cascade of modes into sub-chains but not increase the round-trip phase of either chain. With this knowledge, we can easily calculate that the maximum round-trip phase is obtained by having each mode occur two times - once in both directions - in one large cascade. The total propagation round-trip phase $\Delta\phi_p$ that maximally can be obtained is thus given by the sum of all propagation phases:

$$\Delta\phi_p = 2k_0 L \sum_{i=1}^{N} n_{\mathrm{eff},i}. \tag{S1}$$

Additionally, at each reflection, the wave may experience a nontrivial phase shift. The total maximum round-trip phase is then given by:

$$\Delta\phi = 2k_0 L \sum_{i=1}^{N} n_{\mathrm{eff},i} + 2\sum_{i=1}^{N} \phi_{r,i,i+1}, \tag{S2}$$

where $\phi_{r,i,i+1}$ is the reflection phase upon conversion from mode i to $i+1$. We number the transversal modes in order of appearance in the cascade. In this notation, we implicitly assume that mode $N+1$ is the first mode again.

B. The transmission spectrum of cascaded-mode resonators

In agreement with the traditional derivation of a Fabry-Perot transmission spectrum, we now calculate the transmission spectrum of a general cascaded-mode resonator. We explicitly calculate the partially transmitted fields through the resonator, as the field inside the resonator travels back and forth. We do this in the most general case where N different forward modes are coupled with each other.

In our analysis, we number the modes 1 to N. Because the cyclic nature of the mode conversions, we can rename the first node to coincide with the incident mode and subsequently rename all the other nodes in the order that they appear in the loop.

An incident field is partially transmitted through the resonator, after being transmitted through the first and second reflector and propagating through the cavity. Assuming that the product of the two transmissions through the first and second reflector is given by t_{pt}, we can thus write that:

$$\mathbf{E}_{\mathrm{out},1} = t_{\mathrm{pt}_1} \mathrm{e}^{\mathrm{i}\phi_1} E_{\mathrm{in}} \mathbf{u}_{\mathrm{f}_1}. \tag{S3}$$

The parameter t_{pt_1} encodes the transmission efficiency through both reflectors. The unit vector $\mathbf{u}_{\mathrm{f}1}$ keeps track to the vectorial nature of the field, i.e., the specific mode that is transmitted—in this case forward mode f1.

A significant portion of the field stays inside the resonator and converts to the another mode at the second reflector. After one one backward and one forward propagation of the wave, the second partially transmitted field will be given by

$$\mathbf{E}_{\mathrm{out},2} = t_{\mathrm{pt}_2} \mathrm{e}^{\mathrm{i}\phi_2} E_{\mathrm{in}} \mathbf{u}_{\mathrm{f}_2}. \tag{S4}$$

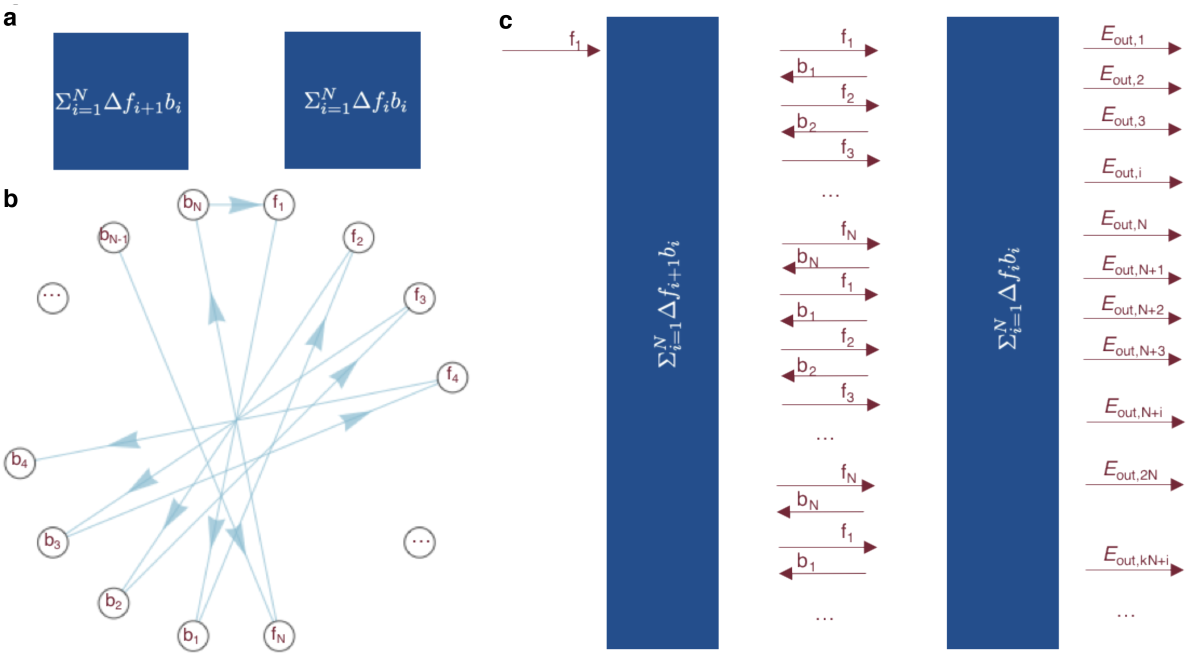

Figure S1. **The derivation of the transmission spectrum of a general cascaded-mode resonator. a,** A compact representation of the mode conversions in the resonator. The modes are renumbered in the order that they appear in the chain, where we distinguish N forward propagating modes (f_i) and N backward propagating modes (b_i). According to this nomenclature, the left and the right reflector implement $\Sigma_{i=1}^{N}\Delta f_{i+1}b_i$ and $\Sigma_{i=1}^{N}\Delta f_i b_i$, respectively. Here, we also assume $\mathrm{f}_{N+1} = \mathrm{f}_1$. **b,** The graph representation of the general resonator, defined in **a**. **c,** A visualization of the partially transmitted fields at the right-hand side of the resonator. The total transmitted field is the infinite sum of all these partially transmitted fields. This sum is given by Eq. (S12).

This picture continues until we have reached the last forward propagating mode in the system, which gives rise to the Nth partially transmitted field:

$$\mathbf{E}_{\mathrm{out},N} = t_{\mathrm{pt}_N}\mathrm{e}^{\mathrm{i}\phi_N}E_{\mathrm{in}}\mathbf{u}_{\mathrm{f}_N}. \tag{S5}$$

After the Nth wave has been partially transmitted, the whole cascade has been completed and the cascade starts again. It is interesting to note that, for each mode transmitted, the following law is now fulfilled:

$$\mathbf{E}_{\mathrm{out},kN+i} = (r_{\mathrm{rt}}\mathrm{e}^{\mathrm{i}\Delta\phi})^k\mathbf{E}_{\mathrm{out},i}, \tag{S6}$$

where i varies between 1 and N and k is an integer number keeping track of the number of full round trips that have been completed.

Here, we retrieve the round-trip phase shift $\Delta\phi$, as defined in the previous section, and we introduce the parameter r_{rt}:

$$r_{\mathrm{rt}} = \prod_{i=1}^{M} r_{i,i+1}, \tag{S7}$$

where M is the total number of mode conversions in one full round trip.

The total transmitted field through the resonator, for a given incident field $\mathbf{E}_{\mathrm{in}}$ is the sum of all the partially

transmitted fields, mathematically taking the sum up to infinity:

$$\mathbf{E}_{\text{out}} = \lim_{m\to\infty} \sum_{j=1}^{m} \mathbf{E}_{\text{out},j} \tag{S8}$$

$$= \lim_{n\to\infty} \sum_{k=0}^{n} \sum_{i=1}^{N} \mathbf{E}_{\text{out},kN+i} \tag{S9}$$

$$= \lim_{n\to\infty} \sum_{i=1}^{N} \sum_{k=0}^{n} \mathbf{E}_{\text{out},kN+i} \tag{S10}$$

$$= \sum_{i=1}^{N} t_{\text{pt}_\text{i}} \mathbf{u}_{\text{f}_i} \mathrm{e}^{\mathrm{i}\phi_i} E_{\text{in}} \lim_{n\to\infty} \sum_{k=0}^{n} (r_{\text{rt}} \mathrm{e}^{\mathrm{i}\Delta\phi})^k. \tag{S11}$$

This last sum is a geometric series. In closed form it can be rewritten as $(1 - r_{\text{rt}}\mathrm{e}^{\mathrm{i}\Delta\phi})^{-1}$. The total transmitted field thus equals:

$$\mathbf{E}_{\text{out}} = \sum_{i=1}^{N} \frac{t_{\text{pt}_i} \mathrm{e}^{\mathrm{i}\phi_i}}{1 - r_{\text{rt}} \mathrm{e}^{\mathrm{i}\Delta\phi}} E_{\text{in}} \mathbf{u}_{\text{f}_i} \tag{S12}$$

In general, for a given incident field, the output will will be a sum of different forward propagating modes, whose amplitudes are given by the different $t_{\text{pt},i}$. The different modes follow the same spectrum, defined by the round-trip reflection efficiency r_{rt} and the round-trip loss $\Delta\phi$.

For each of the output modes, we can calculate the transmitted intensity. Defining $t_i = E_{\text{out},i}/E_{\text{in}}$ and $\mathbf{t}_{\text{pt},i} = t_{\text{pt}_\text{i}} \mathbf{u}_i$, with $\mathbf{u}_i$ the unit vector of output mode i, we get:

$$T_i = t_i^* t_i \tag{S13}$$

$$= \frac{t_{\text{pt}_i}^* t_{\text{pt}_i}}{(1 - r_{\text{rt}}\mathrm{e}^{\mathrm{i}\Delta\phi})(1 - r_{\text{rt}}\mathrm{e}^{-\mathrm{i}\Delta\phi})} \tag{S14}$$

$$= \frac{|t_{\text{pt},i}|^2}{1 - 2r_{\text{rt}}\cos(\Delta\phi) + r_{\text{rt}}^2} \tag{S15}$$

$$= \frac{|t_{\text{pt},i}|^2}{(1 - r_{\text{rt}})^2 + 4r_{\text{rt}}\sin^2(\Delta\phi/2)} \tag{S16}$$

$$= \frac{\alpha_i}{1 + F\sin^2(\Delta\phi/2)}, \tag{S17}$$

where we retrieve a generalized definition of the finesse $F = 4r_{\text{rt}}/(1 - r_{\text{rt}})^2$ and define the normalized outgoing intensity amplitude for mode i: $\alpha_i = |t_{tp_i}|^2/(1 - r_{\text{rt}})^2$.

C. Examples of graphs and adjacency matrices related to cascaded-mode resonators

In Fig. S2, we show the directed graph and the adjacency matrix of three different implementations of cascaded-mode resonators. In (a-c) we present an implementation with $N = 4$ and $\xi = 1$, in (d-f) an implementation with $N = 4$ and $\xi = 2$ and in (g-i) we have a resonator that simultaneously contains two different types of resonances: $N = 4, \xi = 1$ and $N = 1, \xi = 2$.

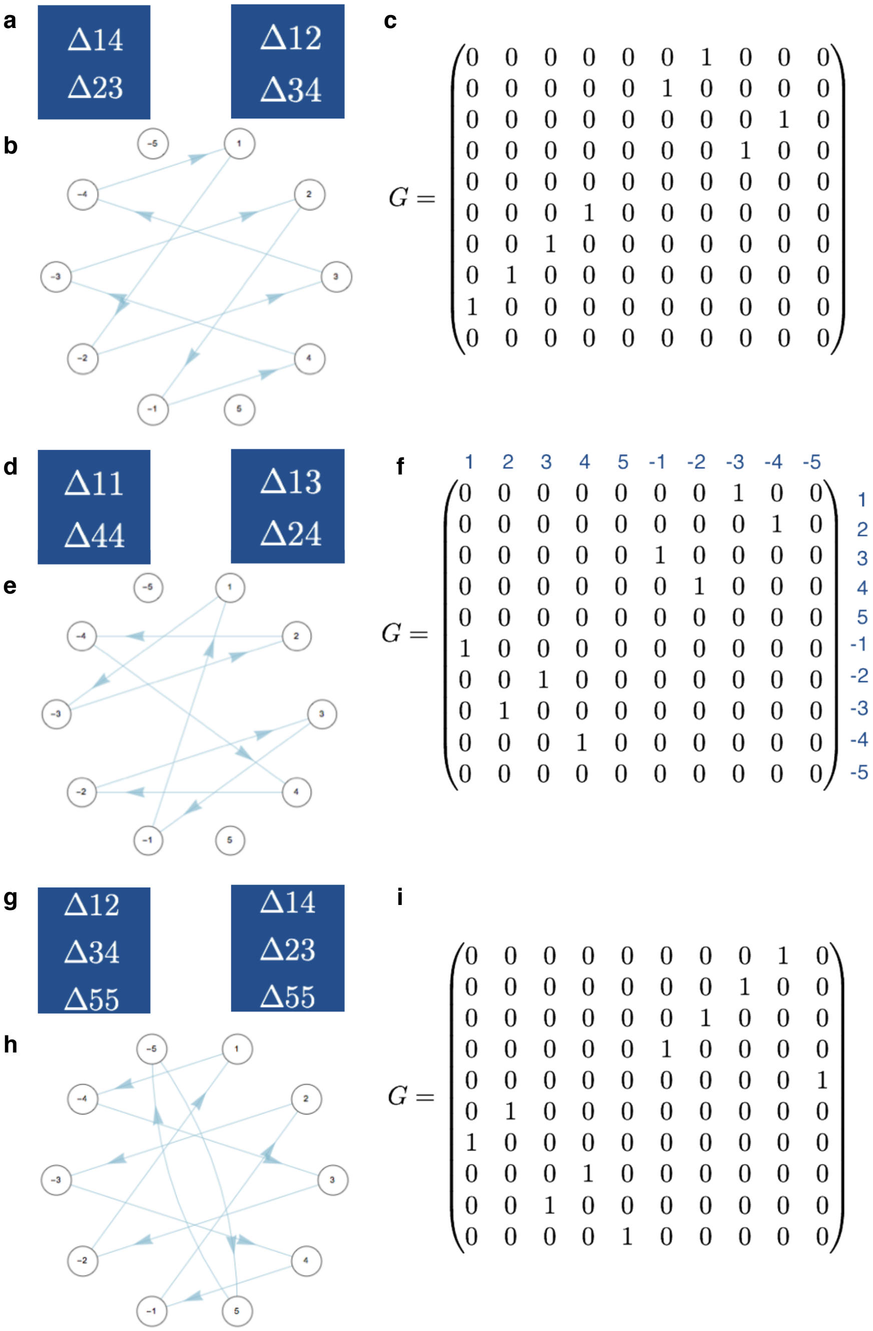

Figure S2. **Several examples of graphs and adjacency matrices related to cascaded-mode resonators. a-c,** A cascaded-mode resonator where $N = 4, \xi = 1$. **d-f,** A cascaded-mode resonator where $N = 4, \xi = 2$. **g-i,** A mixed-resonances cascaded-mode resonator, where two cascaded-mode resonances co-exist: $N = 4, \xi = 1$ and $N = 1, \xi = 2$.

D. Summary of scaling of spectral properties in cascaded-mode resonators

**Spectral parameter**	**traditional resonator (tr)**	**cascaded-mode resonator (CM)**
Free spectral range	$\Delta\nu_{\rm tr}$	$\Delta\nu_{\rm CM} = \Delta\nu_{\rm tr}/N$
Resonance linewidth	$\Delta\gamma_{\rm tr}$	$\Delta\gamma_{\rm CM}/N$
Quality factor	$Q_{\rm tr}$	$Q_{\rm CM} = Q_{\rm tr}N$
Cavity ring-down time	$\tau_{\rm tr}$	$\tau_{\rm CM} = \tau_{\rm tr}N$
Intracavity power build-up	$\kappa_{\rm tr}$	$\kappa_{\rm CM} = \kappa_{\rm tr}N$

II. MATERIALS AND METHODS

A. Fabrication

The integrated photonic circuit structures discussed in this work are fabricated using standard silicon-on-insulator (SOI) fabrication techniques. Starting from an SOI wafer with a 2 μm buried oxide layer and a 220 nm silicon layer, we perform electron-beam lithography with an 125 keV Elionix system using ZEP520A positive resist (spin-coating at 3000 rpm, pre-exposure bake 3 min at 90°C and 3 min at 180°C). After exposure, we develop the photoresist in cold Oxylene for 60 s, and perform an oxygen plasma for 15 s at 40 sccm, 100 W. In a second step, the silicon layer is etched using the resist as an etch mask by single-step reactive ion etching with fluorine chemistry (SF_6 and C_4F_8). The buried oxide layer works as an etch stop layer. The remaining resist layer is then removed by leaving the samples overnight in Remover PG at 80°C. A final cleaning process is performed using Piranha etch for 15 s. Finally, a 700 nm thick cladding layer of silicon dioxide is deposited via chemical vapor deposition.

A set of fabricated samples are shown in Fig. S3 by optical microscope (a) and scanning electron microscope (b-d). Fig S3a illustrates one resonator structure together with waveguides that guide light of a well-defined optical mode (TE_0 or TE_2) into and out of the resonator, which is located in the center of the chip (marked by the square rectangle). This allows to investigate the spectral response of the resonator for a transverse mode of choice. The SEM images in Fig S3b-d illustrate the marked area in the optical microscope image prior to the deposition of the silicon dioxide cladding layer for the three different types of resonators discussed in the main text: (b) the mode converting resonator where upon each reflection at the Bragg mirror, TE_0 transverse modes are transformed into TE_2 modes, and vice-versa; (c) a standard Fabry-Perot resonator which provides selective reflection to the TE_0 tranverse mode, and no mode conversion occurs; and (d) a second Fabry-Perot resonator which provides selective reflection to the TE_2 tranverse mode, and no mode conversion occurs. Each SEM figure contains an inset that shows a close-up view of the mode converter alone. For each resonator, the two Bragg gratings are identical in the cases discussed here. Fig. S3E illustrates a top-view and a side-view schematic of the mode converters as well as the main dimensions of the chip (per = period). The periods are equal to $\mathrm{per} = 313$ (b), 304 (c), 480 nm (d), the duty cycle of all gratings is 40%, the width of the multimode waveguide is $\mathrm{w_{wg}} = 1.07$ μm, and the depth of the corrugations is $\mathrm{D} = 506$ (b), 296 (c), 149 nm (d).

B. Characterization setup

All resonators were characterized by transmission spectroscopy using a tunable Santec TSL-550 laser, having a linewidth of 200 kHz, much below the linewidth of the resonances considered here. The polarization of the incident light is adjusted using fiber polarizers to maximize the power transmitted through the chip. Cleaved fiber probes from Lightwave at an angle of 10° are placed above the grating couplers located at the ends of the on-chip waveguides and couple light from the fiber to on-chip waveguides into TE_0 mode (in-plane polarization). The transmitted power is measured with an InGaAs photodiode with adjustable gain.

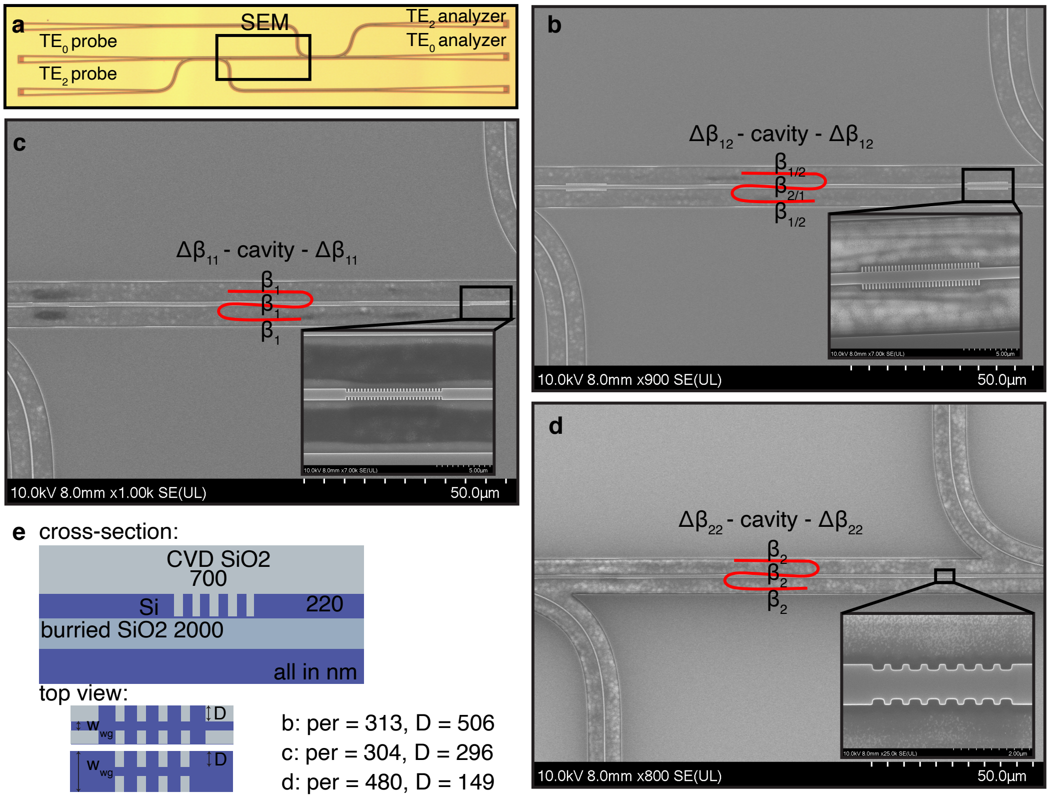

Figure S3. **Images of the fabricated resonators.** **a,** Optical microscope picture of a fabricated resonator features three waveguide inputs on the left and three waveguide outputs on the right. From these, two sets are used to probe the resonator for input TE_0 and TE_2 modes and to analyze the output of the resonator also in these two transverse modes. **b-d,** SEM figures are provided for all three types of resonators we investigate in this work, and a close-up of the Bragg gratings is provided in each figure as an inset. **e,** Top-view and side-view schematics are provided for both types of Bragg reflectors. CVD = chemical vapor deposition, per = period, SiO_2 = silicon dioxide, $\Delta\beta_{11}$ = Bragg mirror that reflects selectively mode TE_0 into TE_0, $\Delta\beta_{12}$ = Bragg mirror that reflects mode TE_0 into TE_2, and vice-versa, $\Delta\beta_{22}$ = Bragg mirror that reflects selectively mode TE_2 into TE_2.

C. Extraction of experimental resonator parameters

In Fig. 4 of the main text, we report resonator parameters (resonant wavelength, quality factor, group index) that were extracted from the experimentally measured transmission spectra. In this section, we elaborate our procedure to extract these parameters, which was applied to all data. We start by fitting Lorentzian lineshapes to each longitudinal mode and use the Lorentzian fit to determine the resonant wavelength and the Q-factor. These are reported in panels a and b. For the computation of the group index from the transmission spectra, we need an accurate estimation of the effective cavity length for each resonator. The effective cavity length $L_{eff} = L_{wg} + 2L_{Bragg}$ accounts for the penetration of optical fields into the Bragg reflectors (which we summarize into an effective Bragg length L_{Bragg}), which adds to the geometrical length of the multimode waveguide L_{wg}. To determine L_{Bragg} experimentally, we fabricated in each case three separate resonators for each type of mode-coverters, each with a length of the multimode waveguide of $L_{wg} = 100\ \mu m$, 150 μm and 200 μm. Since in all three cases, L_{Bragg} is constant, it is possible to extract L_{Bragg} from the free spectral range of the longitudinal modes at different waveguide lengths. We find that $L_{Bragg} = 4.3\ \mu m, 6.15\ \mu m$ and $0.5\ \mu m$ for the mode-converting grating, the TE_0 standard Fabry Perot resonator and the TE_2 standard Fabry Perot resonator. By knowing the effective total cavity length, the group

indices can be extracted from the spacing of adjacent longitudinal modes.

III. SIMULATIONS AND EXPERIMENTS

The multimode waveguide preceding the resonators, located in between the Bragg gratings and after the resonator has a width of $w_{wg} = 1.07\ \mu m$. This width was chosen as to maximize the difference between the effective indices of the TE_0 and TE_2, as to ensure a high selectivity of the three different Bragg mirrors we consider. The simulated effective indices of the modes are reported for various waveguide widths in Fig. S4a. Additionally, the TE_3 mode and all subsequent higher order modes are not supported at this waveguide width. Furthermore, we use adiabatic tapers connected to single-mode waveguides of width 440 nm to filter out any TE_2 mode from the TE_0 analyzer port. The efficiency of the taper and single-mode waveguide to achieve this filtering effect is simulated using finite-time-domain methods and the resulting field distribution is shown in Fig. S4b. As visible from the inset, the transmission is below 10^{-5}.

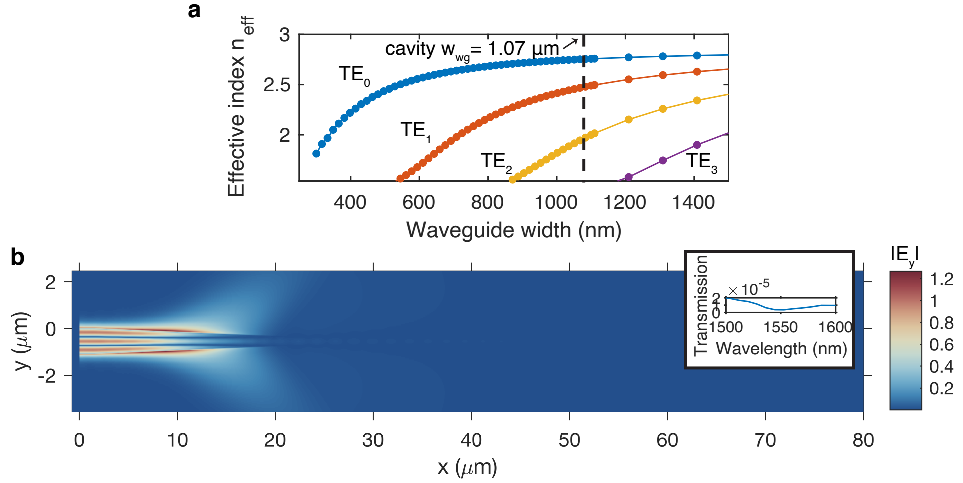

Figure S4. **Multimode waveguide properties.** **a,** Dispersion curves of the effective indices of transverse modes TE_i as a function of waveguide width. **b,** Simulation of transmitted electric field of mode TE_2 through the adiabatic taper reveals a transmission below 10^{-5}.

A. Design of co-directional waveguide coupler

In order to probe and analyze the resonator properties under an incident TE_2, we design a co-directional mode converter based on the evanescent coupling of a nanowaveguide and our multimode waveguide shown in Fig. S5. For the conversion to be efficient, the effective index of the TE_0 mode in the narrow waveguide needs to match the effective index of the TE_2 mode in the multimode waveguide. In this situation, the propagation constants of the two modes are equal and coherent injection of mode TE_2 from TE_0 can be ensured. Graphically, this corresponds to horizontal lines in the plot of Fig. S4a, and to a width of the nanowaveguide of 335 nm. We find from full-wave simulations that a coupling length of $70\ \mu m$ as was used in the experiments, is sufficient to couple 70% of the power into mode TE_2 around a central wavelength of 1550 nm.

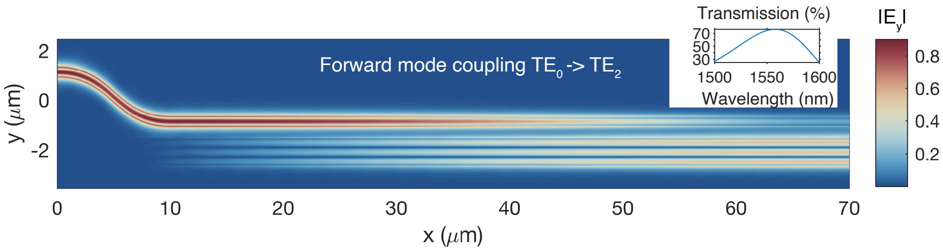

Figure S5. **Co-directional waveguide coupler properties.** Simulation of the adiabatic forward mode coupler shows efficient energy transfer from the narrow single-mode waveguide populated with TE_0 to the wide multimode waveguide where TE_2 is parametrically generated. Inset: A maximal transmission of 70% is achieved.

B. Design of contra-directional mode-converting and non-mode converting Bragg gratings

In this section we discuss the design strategy of mode-converting and non-mode converting Bragg gratings. For these gratings to fulfill their intended purpose, several conditions need to be satisfied concomitantly: 1. all gratings provide selective and efficient reflection for a pre-selected transverse mode profile, 2. all gratings provide only minimal reflection to all other transverse mode profiles, and 3. all gratings effect only negligible co-directional mode conversion. As we anticipate above, these conditions can be fulfilled by careful choice of the effective refractive index of the transverse modes supported by the waveguide. In general, a Bragg grating provides an additional momentum that can be leveraged to achieve phase matching for a given set of modes. For contra-directional coupling of modes with propagation constants $\beta_1 = \mathrm{n}_{\mathrm{eff},1}\frac{\omega}{\mathrm{c}_0}$, and $\beta_2 = \mathrm{n}_{\mathrm{eff},2}\frac{\omega}{\mathrm{c}_0}$ (corresponding to mode-converting Bragg mirrors), the grating period Λ needs to be chosen such that $\frac{2\pi}{\Lambda} = \beta_1 + \beta_2$. For co-directional coupling, the grating period Λ needs to be chosen such that $\frac{2\pi}{\Lambda} = \beta_1 - \beta_2$. We recognize here already that the corresponding grating periods will be considerably distinct and that thereby, in general, the co-directional coupling will be negligible when contra-directional coupling is achieved. For contra-directional coupling of modes with equal propagation constants $\beta_1 = \mathrm{n}_{\mathrm{eff},1}\frac{\omega}{\mathrm{c}_0}$, or $\beta_2 = \mathrm{n}_{\mathrm{eff},2}\frac{\omega}{\mathrm{c}_0}$ (corresponding to mode-converting Bragg mirrors), the grating period Λ needs to be chosen such that $\frac{2\pi}{\Lambda} = 2\beta_1$ or $2\beta_2$ (corresponding to standard non-mode converting Bragg mirrors). Having chosen the two modes TE_0 and TE_2 to have maximally different effective refractive indices, it is possible to choose a grating period that achieves only mode-converting reflection and negligible standard reflection, or vice-versa.

In Fig. S6, we report the simulated reflection and transmission properties of the mode converting grating with parameters as described above, under an incident TE_0 mode. We find that an efficient mode conversion is provided by the grating around 1560 nm, and that reflection into the same mode is negligible. Furthermore, with an increasing number of grating periods, the reflected power at the grating increases.

In Fig. S7, we report the simulated reflection and transmission properties of the mode converting grating with parameters as described above, under an incident TE_2 mode. We find that an efficient mode conversion is provided by the grating around 1560 nm, and that reflection into the same mode is negligible. Furthermore, with an increasing number of grating periods, the reflected power at the grating increases. By comparing panel c of Fig. S6 with panel d of Fig. S7, we observe the reciprocal behavior of the mode converters.

Finally, we report in Fig. S8 the reflection curves for all types of Bragg grating, mode-converting (a) and standard non-mode converting (b,c).

C. Cascaded-mode resonators and Fabry-Perot resonators

Cascaded-mode resonators as discussed in the main text provide resonant confinement to input modes that correspond to either TE_0 or TE_2 transverse modes and have the same transmission spectrum for either input. We contrast here this property with two test Fabry-Perot resonators that employ standard mirrors and provide cavity confinement to only one of TE_0 or TE_2 modes. The experimental results are shown for the three cases in Fig. S9:

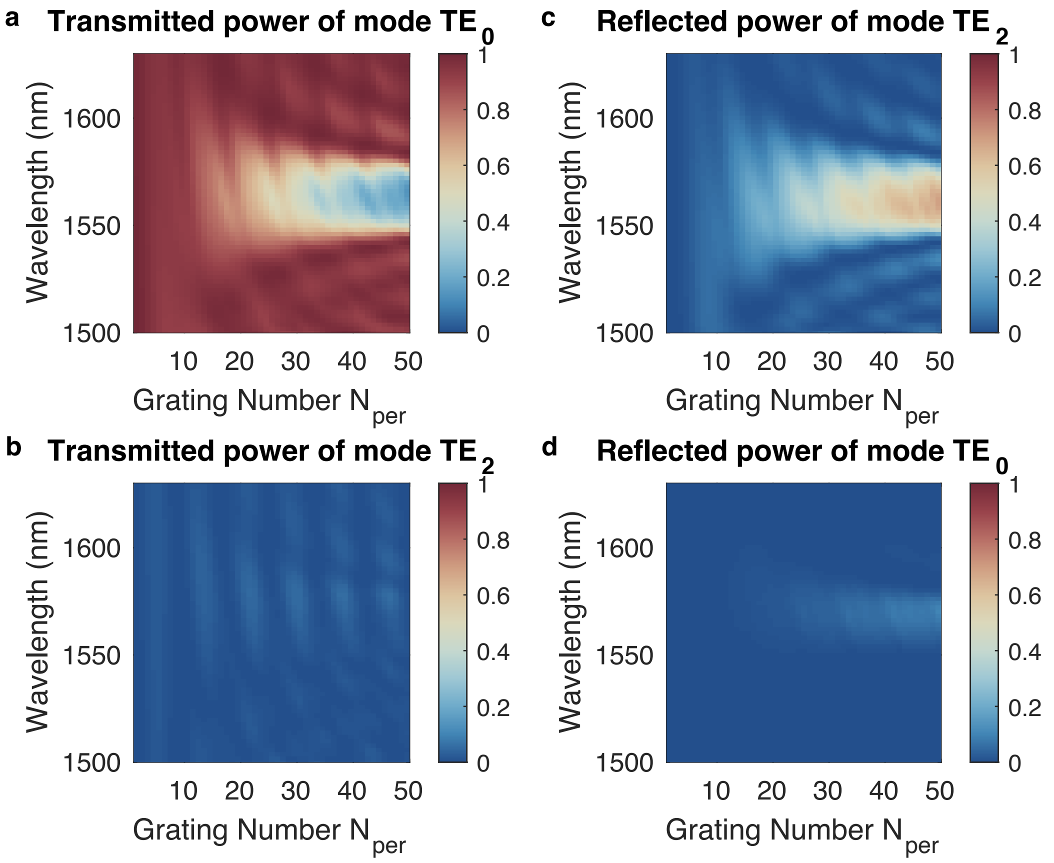

Figure S6. **Contra-directional mode-converting grating properties under an incident** TE_0 **mode. a-b,** Transmitted power into mode TE_0 and TE_2. (**c-d,**) Reflected power into mode TE_0 and TE_2. Overall, these graphs demonstrate an efficient and selective conversion from input mode TE_0 into an output mode with transverse profile TE_2, and that this conversion is efficient in a reflection geometry around 1560 nm. Furthermore, the Bragg grating reflects back into the same mode TE_0 only minimally, and also the co-directional conversion is negligible. Furthermore, as the number of grating periods increases, the reflected power also increases. We chose a number of periods $\mathrm{N}_{\mathrm{per}} = 36$.

cascaded-mode resonator (measurements d-e, simulations f-g), Fabry-Perot resonator operating on the TE_0 mode (h-i) and Fabry-Perot resonator operating on the TE_2 mode (j-k). By comparing the three cases, we find that cavity modes appear, as expected, for both TE_0 and TE_2 modes in the case of the cascaded-mode resonator only. Moreover, the experimental results are well-reproduced by our simulations. Cavity modes appear only for one of the two modes for the conventional Fabry-Perot resonators, while light is simply transmitted for the other modes.

D. The extraction of the experimental resonator parameters

All parameters of the resonators are extracted directly from the measured transmission data presented in Fig. 4b. We fit each individual peak in the transmission (each cascaded-mode resonance) with a Lorentzian lineshape, which is justified given that the residual reflectivity of our reflectors is well below 0.1 (the level of residual reflection

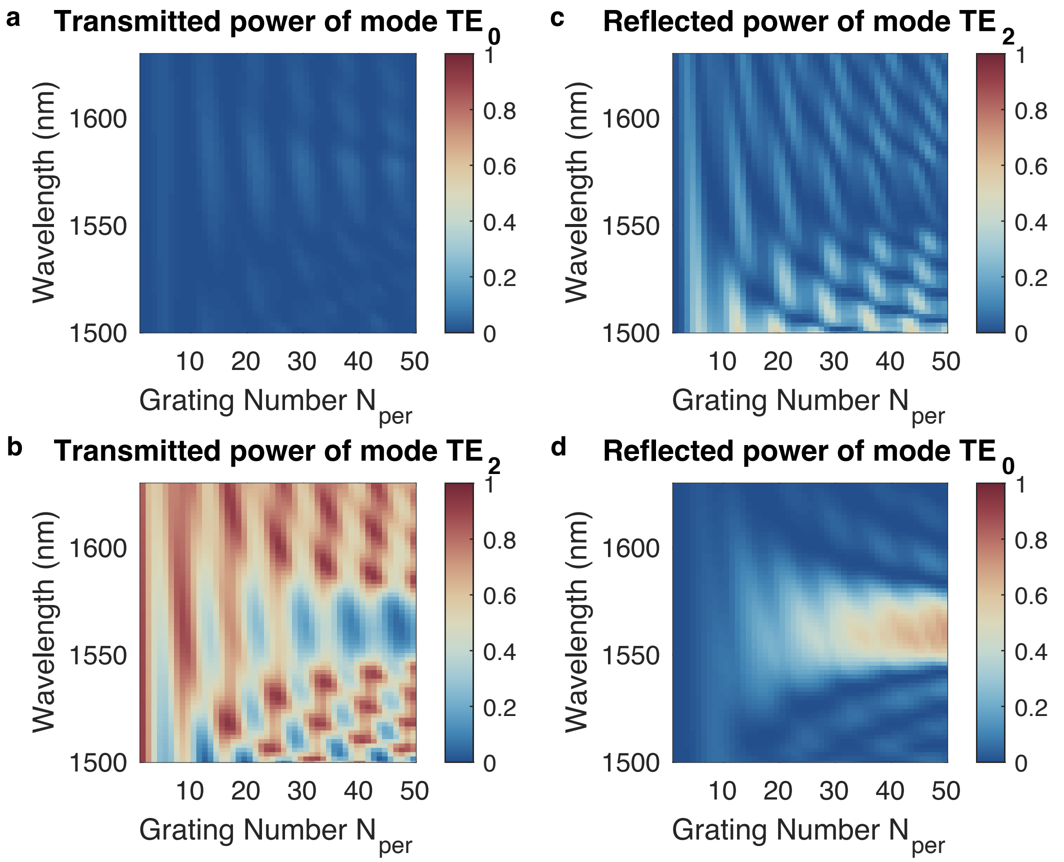

Figure S7. **Contra-directional mode converting grating properties under an incident** TE_2 **mode. a-b,** Transmitted power into mode TE_0 and TE_2. **c-d,** Reflected power into mode TE_0 and TE_2. Overall, these graphs demonstrate an efficient and selective conversion from input mode TE_2 into the output mode with transverse profile TE_0, and that this conversion is efficient in a reflection geometry around 1560 nm. Furthermore, the Bragg grating reflects back into the same mode TE_2 only minimally, and also the co-directional conversion is negligible. Furthermore, as the number of grating periods increases, the reflected power also increases. We chose a number of periods $\mathrm{N}_{\mathrm{per}} = 36$.

where hybridization starts to become visible in the spectrum, see Extended Data Fig. 4). The fit yields a resonance frequency (which is used to report the resonant wavelength in Fig. 4d and the difference between the resonant wavelengths in the upper panel of Fig. 4d) and the linewidth (which is used, together with the resonant wavelength to report the quality factor in Fig. 4e). Finally, the group index is reported in Fig. 4f from the free-spectral range as

$$n_g(\lambda) = \frac{c}{2L_{\mathrm{eff}}\Delta f_{\mathrm{FSR}}} = \frac{\lambda^2}{2L_{\mathrm{eff}}\Delta\lambda_{\mathrm{FSR}}}. \tag{S18}$$

Here, $L_{\mathrm{eff}} = L_{\mathrm{WG}} + 2L_{\mathrm{mirror}}$ is the effective length of the cavity, composed of the geometric length of the multimode waveguide L_{WG} and the penetration length L_{mirror} of the fields into the Bragg grating. The latter depends on the strength of the grating and has been determined experimentally by measuring two different resonators with two different but known lengths of the multimode waveguide.

The error bars in Fig. 4d are the errors that we attribute to the fitting of the resonant wavelength, computed as the mean deviation of the resonant wavelength fitted by the Lorentzian lineshape from the resonant wavelength as

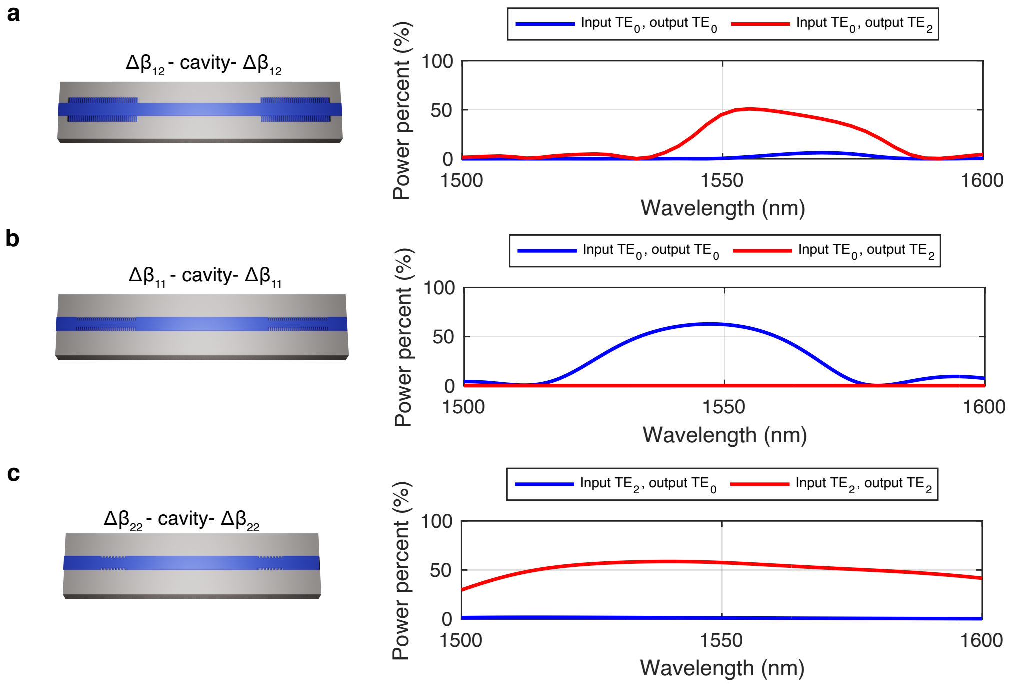

Figure S8. **Reflection properties of mode-converting and non-mode converting gratings. a-c,** The reflection curves of the various Bragg gratings used in the resonators shown in the left panels are reported under different input and output conditions.

found with a peak finder algorithm. This error then accounts for systematic errors in our fitting procedure. The error on the group index in Fig. 4e and 4f, respectively, are the propagated errors assuming that all other physical quantities (e.g., the linewidth) are error-less.

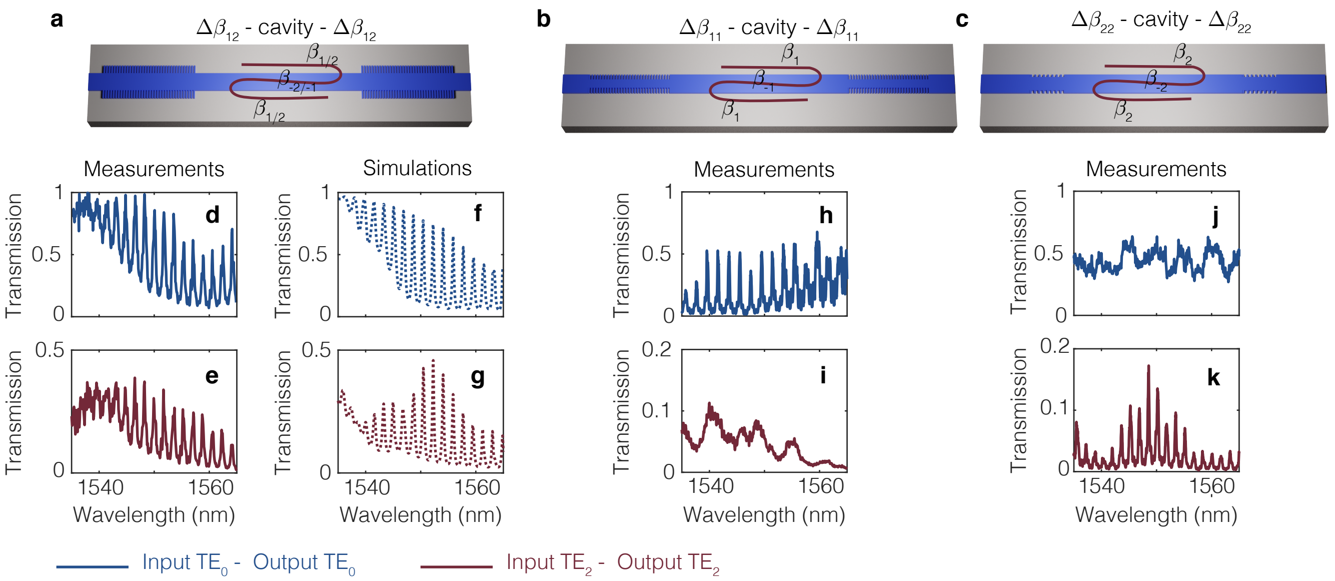

Figure S9. **Transmission spectroscopy of cascaded-mode resonators versus conventional Fabry-Perot resonators. a,** A cascaded-mode resonator, where a reflection at both the left and the right Bragg mirror results into a conversion of the transverse mode from TE_0 to TE_2 and vice-versa, is compared to conventional Fabry-Perot resonators, where no mode conversion occurs upon reflection. Two types of Fabry-Perot resonators are considered, where the Bragg mirror provides selective reflection to either mode TE_0 **b,** or mode TE_2 **c**. **d-e,** The measured transmission spectra of the cascaded-mode resonator exhibit resonances regardless of whether TE_0 or TE_2 is incident onto the resonator. These mode-independent resonances are a defining feature of cascaded-mode resonators. **f-g,** Simulated transmission spectra reproduce well the measurements. **h-i,** Measured transmission of a Fabry-Perot resonator for the case that either TE_0 or TE_2 is incident onto the resonator. The Bragg mirrors reflect selectively only TE_0 and no mode conversion occurs. Cavity modes appear only for the TE_0 input. **j-k,** In this case, the Bragg mirrors only reflect TE_2 and no mode conversion occurs. Cavity modes appear only for the TE_2 input. In all measurements and simulations, the analyzed mode (output mode) is the same as the probe mode (input mode) and the transmission curves are normalized to the transmitted intensity of a bare waveguide without any resonator. Blue curves are transmission measurements under a TE_0 probe/analyzed mode, whereas red curves are transmission measurements under a TE_2 probe/analyzed mode.
